# Occupancy Classification of Position Weight Matrix-Inferred Transcription Factor Binding Sites

**DOI:** 10.1371/journal.pone.0026160

**Published:** 2011-11-04

**Authors:** Hollis Wright, Aaron Cohen, Kemal Sönmez, Gregory Yochum, Shannon McWeeney

**Affiliations:** 1 Division of Bioinformatics and Computational Biology, Department of Medical Informatics and Clinical Epidemiology, Oregon Health and Science University, Portland, Oregon, United States of America; 2 Department of Biochemistry and Molecular Biology, The Pennsylvania State University College of Medicine, Hershey, Pennsylvania, United States of America; 3 Division of Biostatistics, Department of Public Health and Preventive Medicine, Oregon Health and Science University, Portland, Oregon, United States of America; 4 Oregon Clinical and Translational Research Institute, Oregon Health and Science University, Portland, Oregon, United States of America; 5 OHSU Knight Cancer Institute, Oregon Health and Science University, Portland, Oregon, United States of America; University of Vermont, United States of America

## Abstract

**Background:**

Computational prediction of Transcription Factor Binding Sites (TFBS) from sequence data alone is difficult and error-prone. Machine learning techniques utilizing additional environmental information about a predicted binding site (such as distances from the site to particular chromatin features) to determine its occupancy/functionality class show promise as methods to achieve more accurate prediction of true TFBS *in silico*. We evaluate the Bayesian Network (BN) and Support Vector Machine (SVM) machine learning techniques on four distinct TFBS data sets and analyze their performance. We describe the features that are most useful for classification and contrast and compare these feature sets between the factors.

**Results:**

Our results demonstrate good performance of classifiers both on TFBS for transcription factors used for initial training and for TFBS for other factors in cross-classification experiments. We find that distances to chromatin modifications (specifically, histone modification islands) as well as distances between such modifications to be effective predictors of TFBS occupancy, though the impact of individual predictors is largely TF specific. In our experiments, Bayesian network classifiers outperform SVM classifiers.

**Conclusions:**

Our results demonstrate good performance of machine learning techniques on the problem of occupancy classification, and demonstrate that effective classification can be achieved using distances to chromatin features. We additionally demonstrate that cross-classification of TFBS is possible, suggesting the possibility of constructing a generalizable occupancy classifier capable of handling TFBS for many different transcription factors.

## Introduction

Computational discovery of Transcription Factor Binding Sites (TFBS) is a difficult problem. Both methods utilizing Position-Weight Matrices (PWMs, [1], [2]) and methods using ab initio prediction (e.g. MEME [3]), Weeder [4]) show high error rates in prediction of binding sites ([5], [6]). However, methods of identifying TFBS which utilize additional genomic information beyond the DNA sequence have been developed in recent years. Examples of these methods include PhyME [7], which utilizes phylogenetic information as part of an ab initio motif discovery process, and the approach of Chen et al. [8], which uses a Bayesian Network (BN) [9], [10] as a classifier and a number of chromatin features as predictors to classify the expected physical occupancy of PWM-predicted TFBS. The latter paper typifies what we term an “occupancy classification” approach to the problem of identifying high-occupancy or “true” TFBS, in which machine learning techniques integrate information from multiple data sources to predict the occupancy and putative functionality of a given predicted binding site. Techniques that have incorporated additional genomic landscape information have shown improvement in performance over purely sequence-based techniques; however, an evaluation of the applicability of such techniques using multiple machine learning methods and multiple transcription factors has been lacking. In this paper, we present a novel classification approach utilizing the occupancy classification paradigm and a variety of potential predicting features including histone modifications and DNA hypomethylation. We then analyze its performance using multiple machine learning methods as classifiers and multiple publicly available chromatin immunoprecipitation (ChIP)-based transcription factor (TF) binding data sets as training and test data set.

## Methods

**Figure 1:**
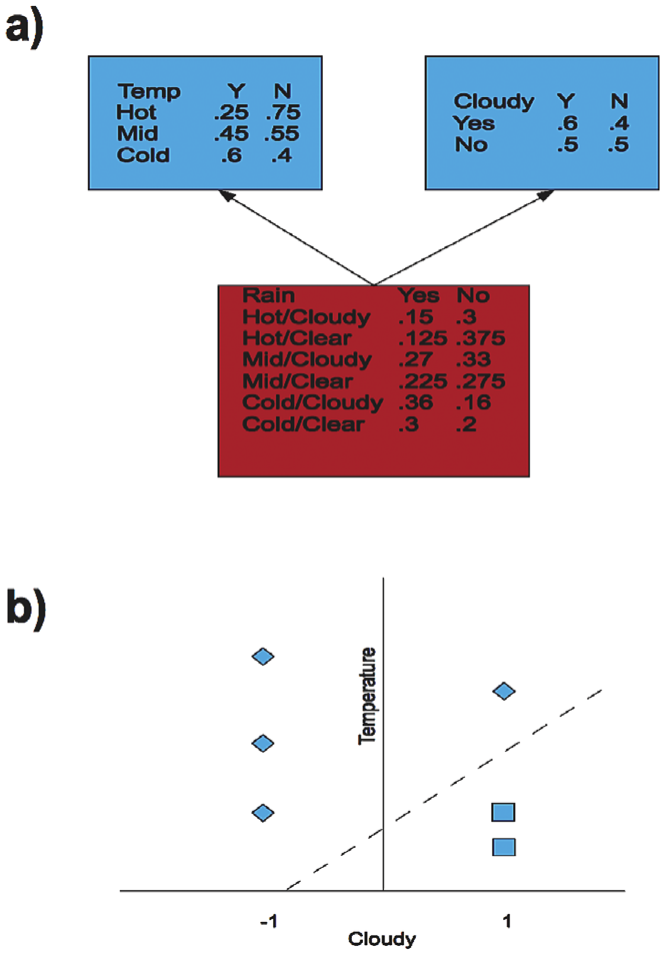
Examples of Classifiers Used in These Experiments. Figure 1a:**Simple Example of Bayesian Network.** Simple Bayesian Network for predicting rain (outcome variable, red) based on temperature and cloud cover (predictor variables, blue). The structure of the network indicates that the probabilities of the predictor variables are independently used to predict the outcome (“Naïve” Bayesian). Probabilities for each outcome given the state of the predictor are given in each predictor node, while the joint probability for each combination is given in the outcome box. The probability of a given outcome can then be calculated based on the joint probabilities given the state of the predictor variables and the prior probabilities of the outcomes. For example, assume cloudy skies and a hot temperature, and that the prior probabilities of rain/not rain are each .5, In this case, the prior probabilities cancel out and the conditional probability (P(rain)|Hot&Cloudy) equals (.15/(.15+.3)) = .33, and P(not rain|Hot&Cloudy) equals (.3/(.15+.3)) = .67. A Bayesian network classifier would therefore predict no rain. Figure 1b:**Simple Example of Support Vector Machine.** Simple Support Vector Machine for predicting rain given temperature and cloud cover, as in Figure 1a. Temperature is represented on the vertical axis, while cloud cover has been dichotomized (−1 = clear, 1 = cloudy). Clear instances are represented diamonds, while cloudy instances are represented by squares. The separating hyperplane is the dotted line, calculated as a combination of a subset of the training data points (support vectors). An instance to be classified that maps to the space above the hyperplane would be predicted to have no rain (e.g. high temperature, not cloudy), while those mapping below the hyperplane would be predicted to be rainy (e.g. low temperature, cloudy). In this ideal case, the hyperplane cleanly separates the classes; however, in a case where this would not be possible (e.g., a hot, clear, rainy day in the training data), the classifier attempts to construct a hyperplane that minimizes the error rate of the classifier.

**Figure 2:**
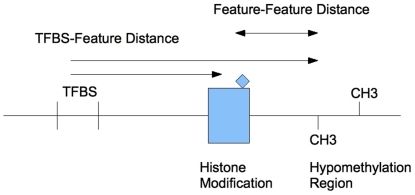
Illustration of TFBS-feature and feature-feature distances. Distances from a predicted TFBS site to the nearest example of a particular histone modification or hypomethylation region are TFBS-feature distances, while the distance from the hypomethylation region or histone modification to the nearest feature of another type are feature-feature distances. Note that these need not be the features used to calculate the feature-feature distance, though this is the case in the figure for clarity.

We identified regions of the human genome on chromosomes 1–22 found to bind a TF according to chromatin immunopreciptation for the factors c-Myc [11], TCF4/TCF7L2 [12], STAT1 [13], GABP and SRF [14]. These regions were mapped to the UCSC Genome Database hg18 build of the human genome ([15], [16]), using a custom MySQL database (MySQL AB). We chose these TFs because each had high-quality whole-genome datasets available and all are thought to function primarily as transcriptional activators, either individually ([17], [18], [19]), or in the beta-catenin/TCF4 complex in the case of TCF4 [20]. In addition, at least three of these factors have known effects in transcriptional pathways important to human health and disease; mutation of c-Myc and/or TCF4 in the Wnt pathway has a driving role in carcinogenesis in many colon cancers [21], while STAT1 is involved in JAK-STAT signalling and interferon response [18]. SRF was held out for later analysis as a blinded TF. TRANSFAC [22] PWMs for the factors were used to predict TFBS in the genome, using the TFBS Perl package [23] and a 95% similarity threshold; the scope of the analysis was limited to regions within +/−3 kb of an annotated transcription start site (TSS) for the 4 TFs in the analysis. Predicted TFBS within 1 kb of the center of an empirically identified TF binding region (defined as a chromoprecipitation/CHiP-seq “hit” as reported by the authors of the respective study) were considered to be “high-occupancy” TF binding sites, while any other predicted site was considered low-occupancy. For each TF, we constructed ten sample data sets via random selection, each with 200 high-occupancy and an approximately equal number of low-occupancy sites per sample; there was some variation in the number of low-occupancy sites due to randomization. Individual predicted binding sites may appear in multiple sample data sets, but are only represented once in a given sample data set. We then trained two types of classifiers: Bayesian Networks (BN) [9], [10] and Support Vector Machines (SVM) [24]. Bayesian Networks utilize a joint probability distribution of associations of predictor variables with outcomes, utilizing Bayes's rule to calculate the most likely state of the outcome variable given the states of the predictors and thereby perform classification, while Support Vector Machines use a separating hyperplane to separate classes in a feature space. This hyperplane is constructed as a combination of training data points about the hyperplane; these are the “support vectors” (see Figures 1a and 1b). In general, Bayesian networks require “binned” data that has been discretized into individual classes (e.g. near/far rather than a continuous distance measure), while SVMs can handle data that is either binned or continuous. We trained these classifiers in the Weka machine learning environment [25] and utilzing the LibSVM SVM library [26], to discriminate between the sites using a variety of features. The features used were distances to nearest histone modification islands ([27], [28]), nearest hypomethylation island as identified in leukocytes [29], and nearest CpG islands and nearest TSS as identified in the UCSC Genome Browser (hereafter referred to as TFBS-feature distances). We additionally incorporated distances between these nearest chromatin features to the nearest chromatin feature of a given type (hereafter referred to as feature-feature distances); e.g. the distance between a nearest H3K4 trimethylation feature to a TFBS and the nearest CpG island to that H3K4 feature (see Figure 2). Feature-feature distances were capped at a maximum of 10 kb (e.g., a closest distance larger than 10 kb was treated as 10 kb) between features to speed construction of the data sets. The specific classification algorithms used were:

- A BN using the K2 network-building algorithm [29], MDL-based discretization for binning [30], and the CFS-subset algorithm [31] for attribute selection.
- A linear-kernel SVM using default parameters
- A linear-kernel SVM with attributes preprocessed into bins using the same MDL-based discretization technique as in the BN classifiers.

We evaluated the classifiers' ability to discriminate high and low occupancy sites using 10-fold cross-validation of each sample and the area under the curve (AUC) metric; AUC is calculated as the area under a plot of true positive rates and false positive rates for the classifier, with an AUC of 1 indicating perfect classification performance and and area of .5 indicating equivalence to randomly assigning classes to test examples (i.e., no effective classification ability). We compared the algorithm and feature sets used for the best-performing classifier for each TF. For each TF, we also constructed classifiers on a per chromosome basis as described above, extracting training data from the other chromosomes. We also evaluated the performance of each best classifier on the other TFs in the study, using each sample as a training set and classifying each other sample from the other three TFs. Additionally, we evaluated the difference in performance between the classifiers when the feature-feature distances were excluded from the feature set, using cross-validation and the AUC as described previously. Finally, we performed an analysis of the agreement between TFs on relevant features based on number of times of a feature's inclusion in the cross-validation classifier using Cohen's kappa measure [32] as implemented in the e1071 package for R 2.7 [33], and performed a cross-classification of the held-out SRF data set with each TF to further examine cross-classification performance.

## Results

For the four TFs we analyzed (c-Myc, TCF4, STAT1, and GABP) and features we utilized (distances to histone modifications, DNA hypomethylation, CpG islands and TSS) we found that the Bayesian Network (BN)-based classifiers consistently outperformed Support Vector Machine (SVM)-based classifiers for all TFs, and achieved average AUC scores ranging from .71 (TCF4) to .94 (GABP). Area Under the Curve (AUC) scores achieved in per-chromosome classification were comparable to those achieved in cross-validation. The resultant classifiers have naïve Bayesian network structures. Both TFBS-feature and feature-feature distances are predictive, but the feature-feature distances appeared to be the dominant predictors for all TFs. No features of either type appear to be universally predictive across all TFs. Classification of other TFs by a classifier trained on a different TF was universally inferior to performance of a classifier trained on the TF of interest, excepting the case of SRF. All TFs were capable of accurate cross-classification of the held-out SRF data set.

### Comparison of Algorithms

In all cases, the BN classifier outperformed either of the SVM-based classifiers (see Table 1). This result indicates that binning/discretization of data types does not appear to grant the BN method any advantages relative to the BN; however, the performance advantage of the BN may be a result of a high difficulty of assigning an error-minimizing hyperplane for an SVM, due to overlaps in the feature space between classes. The effect of binning may have been to exacerbate this issue, leading to the lower performance of the SVM classifiers using discretization. The network structures of the BN classifiers were equivalent to naïve Bayesian networks after discretization and attribute selection, with no edges between the predictors despite the use of the K2 algorithm. We attempted to use the Tree-Augmented Naïve Bayes algorithm [34] to induce additional edges between the predictor nodes in the networks, but this resulted in a marginally worse classification performance. Similarly, use of more sophisticated polynomial and radial basis function kernels for the SVM-based classifiers did not improve performance over the linear kernel. This seems to indicate that interaction effects between the predictors do not seem to have predictive value as such for occupancy, at least at the scale of our experiments. Typical size of the networks was on the order of 50 predictors.

**Table 1:** Average AUC score for each classifier and TF (10 data sets, 10-fold cross-validation).

TF	BN	SVM	SVM (Discretized)
c-Myc	0.74	0.71	0.69
TCF4	0.71	0.67	0.66
STAT1	0.83	0.78	0.75
GABP	0.94	0.91	0.9

Table 1 compares the average AUC score of 100 total cross-validation runs from each of the three classification schemes used in these experiments.

### Contribution of Feature-Feature Distances to Classification

A surprising result of the classification experiments was the dominance of feature-feature distances over TFBS-feature distances in the best-performing classifiers. We therefore decided to rerun classification as above using TFBS-feature distances only. For this and all subsequent analyses, we chose to only construct BN classifiers as they had outperformed SVMs previously. In all cases, average AUC of classification improved with the inclusion of feature-feature distances relative to TFBS-feature distances only (see Table 2). As with overall classification performance, it is difficult to determine how much of the variance in improvement is attributable to biological differences in the TFs versus technical differences in the generation of the data sets. In general, however, the gain in performance tends to drop for ChIP-Seq data vs. data generated from other techniques. From a biological standpoint, the gain in performance could be interpreted from the point of view of the “histone code” hypothesis [35], indicating that the arrangement of chromatin features relative to one another influences the occupancy of that potential TFBS.

**Table 2:** Comparison of average AUC between BN classifiers trained on all available features vs. only TFBS-feature distances.

Factor	All	TFBS-Feature Only
c-Myc	0.74	0.72
TCF4	0.71	0.69
STAT1	0.83	0.82
GABP	0.94	0.92

Table 2 indicates the average AUC across 100 cross-validation classifiers constructed using all available predictors vs. the same classifiers when feature-feature data was excluded.

### Individual Chromosome Classification

We additionally constructed BN classifiers using feature-feature distances for each individual chromosome and TF, generating training data from the other chromosomes. Performance was quite comparable on average to the performance achieved in the randomly sampled data sets (Table 3). However, c-Myc and TCF4 showed considerable variance in performance between chromosomes relative to STAT1 and especially GABP, which were more consistent per chromosome (See Table S1).

**Table 3:** Average AUC for per chromosome classification experiments.

TF	Avg. AUC
c-Myc	0.75
GABP	0.94
STAT1	0.83
TCF4	0.83

Table 3 lists the average AUC for per chromosome classifiers, where training data was isolated from all autosomes (save one) and used to train a classifier to classify occupancy of TFBS on the held out chromosome. This is therefore the average across 22 such experiments for each TF.

### Common Predictors Across TFs

To identify whether or not common predictors were shared across the TFs, we performed a frequency count of all predictors which appeared in at least one classifier instance for all four TFs, over all cross-validations.; see Table 4 for top ten such predictors. The degree of concordance between the TFs once again appears to have a relationship with the method of generation of the data sets, with GABP and STAT1 showing relatively higher concordance with one another, using Cohen's kappa calculated over all attributes (average kappa = .47). c-Myc and TCF4 had less concordance with each other or with STAT1 or GABP (highest average kappa between any other combination of TFs was STAT1-TCF4, with kappa = .43, see Table 5 . Additionally, while the other 3 TFs have at least one predictor that is selected in 80+ classifiers, TCF4's most commonly selected feature (distance to TSS) is selected only in 60 classifiers, and was only rarely selected by the other TFs (and, hence, does not appear in the overall ranking presented in Table 4). No specific predictor appears to be universally applicable to all of the TFs in this study. It is notable that 9 of the top 10 most commonly selected predictors involve distances to H3K4me3 modification islands (in particular, the top 3 feature pairs H3K4me2-H3K4me3, H3K27me1-H3K27me3, and TSS-H3K4me3, as well as H4K20me1-H3K4me3), as presence of H3K4me3 (as well as H3K4me2, H3K27me1 and H4K20me1) histone modification islands have been shown to be correlated with higher gene expression levels [36], which is biologically consistent with the generally accepted functions of the TFs in this study. While individual per-predictor probabilities varied depending on training set and subsequent binning, in general assigned probabilities behaved in a way consistent with biological evidence (e.g., closer distances with respect to the H3K4me histone mark usually assigned higher probabilities for high-occupancy to the TFBS site in question). Average class-conditional probabilities for high-occupancy sites in both the cross-classification and per-chromosome classification for the smallest distance bin in the top 10 most frequently occurring predictors are summarized in Tables S2a (cross-classification, two-bin cases only) and S2b (per chromosome, all cases).

**Table 4:** Top 10 most frequently occurring predictors in the occupancy classifiers (per-TF and cumulative, sorted by cumulative frequency).

Predictor	GABP	c-Myc	STAT1	TCF4	Total
H3K4me2-H3K4me3	80	8	83	57	228
H3K27me1-H3K4me3	84	11	87	43	225
TSS-H3K4me3	85	47	50	22	204
H3K79me1-H3K4me3	69	26	79	25	199
H3K79me2-H3K4me3	97	12	55	34	198
H4R3me2-H3K4me3	61	40	74	23	198
H3K79me2-TSS	81	44	51	20	196
H4K20me1-H3K4me3	68	10	82	30	190
H3K79me3-H3K4me3	30	36	72	49	187
H3K9me1-H3K4me3	78	10	77	13	178

Table 4 indicates the number of times a particular predictor variable occurs in each of the 100 Bayesian network classifiers constructed during cross-validation. The table is ordered according to the cumulative occurrence of the predictor variable in all classifiers across the four TFs.

**Table 5:** Pairwise Agreement on Inclusion of Features into Classifiers (Average Kappa, 553 features, n = 100 per feature).

TF 1	TF2	Kappa
GABP	STAT1	0.47
STAT1	TCF4	0.43
GABP	TCF4	0.41
STAT1	c-Myc	0.39
c-Myc	TCF4	0.36
GABP	c-Myc	0.34

Table 5 indicates the Cohen's kappa score for agreement on the number of times a given predictor was included in the 100 cross-vaidation Bayesian networks between two TFs.

### Cross-Classification Performance

We additionally explored the classification performance achieved using one TF's classifier on the data for the other three TFs. Cross-classification performance may both be an indicator of the suitability of one TF for predicting occupancy of another (perhaps for exploratory modelling purposes), as well as a general indicator of the commonality of features influencing occupancy of a TFBS. For this experiment, each sample from the previous experiments was used to train a BN classifier using the entire sample as training data. This classifier was then tested using each sample from each other TF, resulting in 100 AUC values for each TF-TF pair (Table 6). Both TFBS-feature and feature-feature distances were included. In general, the classification performance achieved appears to correspond well with that achieved in the cross-validation experiments. Both STAT1 and GABP achieve cross-classification performance on one another comparable to that achieved in cross-validation; this result is sensible in light of the number of predictors that the two TFs were found to have in common during the predictor frequency analysis.

**Table 6:** Average AUC in cross-classification experiments.

Training/Test TF	c-Myc	TCF4	STAT1	GABP
c-Myc	x	0.64	0.79	0.86
TCF4	0.65	x	0.78	0.91
STAT1	0.69	0.69	x	0.92
GABP	0.67	0.69	0.83	x

Table 6 indicates the average AUC score across ten classifiers where training data from one TF (rows) was used to train a classifier that classified TFBS for another TF (columns).

### Cross-classification of SRF

SRF represents a unique data set relative to the other TFs in that its dataset appears to contain multiple strong motifs that differ from the “canonical” SRF binding sequence; the TRANSFAC SRF PWM accounts for only about 33% of the sites reported by Valouev et al. [14] After restricting to the 3 kb window about the TSS, only 46 high-occupancy and 421 low-occupancy sites were identified. Because of this low sample size, we chose to exclude SRF from the general analyses described above. However, the data set does represent a tractable “use case” scenario for occupancy classification; we hence decided to investigate the cross-classification performance of the classifiers trained on the TFs previously analyzed on the SRF dataset. SRF appears to be highly amenable to cross-classification (Table 7). While the SRF dataset is quite small, these results both indicate that occupancy classification can operate well on datasets with smaller sample size and an imbalance of high and low-occupancy TFBS sites and that the method used to generate a binding data set likely plays an important role in the accuracy of the evaluation of our classifiers, as the SRF data set is a ChIP-Seq data set and classification performance is comparable to that achieved on other ChIP-seq based data sets.

**Table 7:** Average AUC for SRF cross-classification experiments.

Classifier	AUC
SRF (cross-val)	0.88
GABP	0.88
c-Myc	0.86
STAT1	0.89
TCF4	0.86

Table 7 indicates the average AUC score across 10 classifiers where the indicated TF was used to train a classifier that was tested on SRF TFBS.

## Discussion

While we were able to achieve good performance with occupancy classification overall, considerable variation in performance and in predictors was observed between the TFs. Biological differences are likely at play to some extent; for example, GABP is thought to bind at the majority of human bidirectional promoters [37], and this may imply a stronger or more specific dependence on chromatin environment cues relative to the other factors. Conversely, c-Myc has been shown to have considerable binding activity outside of the window of analysis used in these experiments [38], and this result in conjunction with our classification results may suggest that c-Myc TFBS occupancy in general may not be as sensitive to the chromatin environment local to the 5′ region of genes. However, GABP and c-Myc also represent temporal and technical extremes, with c-Myc data generated in 2004 via paired-end ditag techniques vs. GABP data generated in 2008 via ChIP-seq; this illustrates the difficulty of separating technical from biological variation in performance in these results. We suspect that differences between the techniques used to generate the binding data we utilized (particularly with regard to site coverage) explain many of the discrepancies in performance between TFs, and that our performance on e.g. c-Myc would be probably be improved, possibly to a level comparable to that achieved on STAT1 or GABP classification, given a more complete training set. Another difficulty arises from potential differences in the chromatin environment in the cell lines from which binding data was generated versus the cell lines from which histone modification and hypomethylation data was generated; ideally, all of the data for a given TF and predictors would be derived from the same cell lines. However, such data is not to our knowledge publicly available, and in its absence determining the exact contribution of potential cell line factors to the difference in classification performance for each TF is not possible. There is evidence that variation in histone modification proximal to core promoters or a TSS is less pronounced across cell types [14], however, and this suggests that cell line variations may be less likely to have a severe detrimental effect on our classification performance.

In comparison with other work in the field, the analysis presented addresses several unanswered questions about occupancy classification, notably the question of whether or not accurate cross-classification of one TF by another is possible. The most directly comparable work is that of Chen et al. [8], who constructed a c-Myc classifier using a Bayesian network and distances to various DNA and chromatin features as well as sequence conservation. Chen et al. do attempt to address the issue of cross-classification by cross-classifying CREB binding sites using their c-Myc classifier, but do not address the issue of algorithm comparison in any capacity. The analysis presented in this work addresses more TFs then Chen et al. as well as comparing two distinct algorithms for classification. Our work also addresses the issue of cross-classification of TFs in considerably greater depth. Two additional novel features separate this analysis from that of Chen et al.; the construction of our data sets from raw binding data and the use of feature-feature distances in the classifiers. Chen et al. use as a training data set binding data that quantitatively identifies the level of binding at several c-Myc binding sites. In contrast, this analysis uses only binding data that is not identified beyond presence/absence, and yet achieves reasonable performance, demonstrating that quantitative binding information is not a prerequisite for training accurate occupancy classifiers. Also, the use of feature-feature distances is to our knowledge unique for purposes of identifying high occupancy TFBS, and is not present in Chen et al.

A more recent work is that of Won, Ren, and Wang [39], which uses a Hidden Markov Model-based approach to accurately identify binding sites for 13 distinct TFs in mouse. Our methods and analyses do share important common features, notably the reliance on histone modifications as primary inputs to the classifier, indicating independently (and in agreement with previous evidence such as Chen et al.) that histone modifications are important predictors for occupancy classification in general. The approach of Won, Ren, and Wang (called “Chromia”) has clear distinctions from the analysis presented here and is able to address enhancer regions that our method does not currently attempt to address. We do note though their model loses some performance in enhancers as compared to promoters. However, there are important issues that our methods and analysis address that theirs does not, providing a clear utility to our approach. Won, Ren and Wang do not address the use of feature-feature distances in any fashion, which our approach does examine. The method presented in this work is agnostic to the motif or method used to identify potential TFBS, whereas the identification of TFBS by a TF-specific motif is intimately tied into the model used by Chromia. Won, Ren and Wang do not address the issue of cross-classification in their paper, and indeed Chromia may not be able to perform cross-classification accurately or at all given the necessity of a TF-specific motif in its model. This considerably reduces the method's potential for use in situations where training data may be sparse or unavailable. The separation of TFBS identification from occupancy classification, in addition to enabling us to perform accurate cross-classification, may provide our methods the ability to generalize onto any given TFBS discovery algorithm. We speculate this may be useful in generalizing occupancy classification for situations in which training data for a TF is not available or inadequate or to tailoring occupancy classification to specific needs (e.g., use of ab initio prediction types in lieu of PWM prediction if PWMs are unavailable for a factor of interest).

We believe we have demonstrated the viability of occupancy classification as a method of accurately locating likely high-occupancy TFBS for multiple TFs, and these results suggest a multitude of future research directions for refining and expanding upon occupancy classification methods. An analysis of the impact of using occupancy classification as a supplement to or in lieu of biological data in a real-world analysis of a biological problem is still wanting, but we have begun to design such an analysis based on utilizing protein-protein interaction (PPI) network construction and the occupancy classification methodology that we have described here. Additionally, given the results of both the cross-validation and cross-classification experiments, it seems possible that a combination of occupancy classifiers trained on data from several TFs (possibly through a voting or stacking [40] mechanism) may be able to perform accurate occupancy classification for a variety of different TFs, potentially without requiring explicit training on biological examples for a novel TF. Whether such a classifier is possible and to what degree it would be similar to the classifiers built for this paper are open questions; as an example, it is conceivable that separate generalizable classifiers for repressive transcription factors may be required as opposed to the activating factors used in our experiments, or that performance might be enhanced by using TFs closely related to a novel factor of interest. Another open question available for further study is the construction of an accurate classifier for TFBS located in alternate genomic contexts such as the 3′ regions of genes. However, this work provides key initial steps in that direction.

## Supporting Information

Table S1 **Per-chromosome AUC, true positive rate, true negative rate, and High and Low Occupancy Site Count for all TFs.** Table S1a: c-Myc. Table S1b: GABP. Table S1c: STAT. Table S1d: TCF4.
(DOC)

Table S2 A: Top 10 most frequently occurring predictors in the occupancy classifiers (per-TF and cumulative). B: Average class-conditional probability of high-occupancy status for smallest distance bin for top 10 most frequently occurring features in cross-classification (two-bin cases only). C: Average class-conditional probability of high-occupancy status for smallest distance bin for top 10 most frequently occurring features per-chromosome(all cases).
(DOC)
