# Supplementary material for: Occupancy Classification of Position Weight Matrix-Inferred Transcription Factor Binding Sites
Source: PLoS One. 2011 Nov 4;6(11):e26160. doi: 10.1371/journal.pone.0026160 (PMC3208542; doi:10.1371/journal.pone.0026160)
Supplement: Table S1 — Per-chromosome AUC, true positive rate, true negative rate, and High and Low Occupancy Site Count for all TFs. Table S1a: c-Myc. Table S1b: GABP. Table S1c: STAT. Table S1d: TCF4. (DOC) [file pone.0026160.s001.doc]

Supplementary Material

Table S1: Per-chromosome AUC, true positive rate, true negative rate, and High and Low Occupancy Site Count for all TFs

Table S1a

| Chromosome | AUC | TP | TN | High | Low |
| --- | --- | --- | --- | --- | --- |
| 1 | 0.86 | 0.7 | 0.96 | 47 | 5115 |
| 2 | 0.83 | 0.77 | 0.83 | 13 | 3399 |
| 3 | 0.84 | 0.52 | 0.89 | 21 | 2679 |
| 4 | 0.73 | 0.5 | 0.84 | 10 | 1771 |
| 5 | 0.81 | 0.33 | 0.88 | 9 | 2360 |
| 6 | 0.86 | 0.82 | 0.84 | 11 | 2270 |
| 7 | 0.64 | 0.33 | 0.92 | 18 | 2911 |
| 8 | 0.82 | 0.43 | 0.84 | 14 | 1997 |
| 9 | 0.79 | 0.72 | 0.73 | 39 | 2504 |
| 10 | 0.96 | 0.8 | 1 | 10 | 2471 |
| 11 | 0.88 | 0.82 | 0.78 | 33 | 3174 |
| 12 | 0.79 | 0.74 | 0.84 | 65 | 2651 |
| 13 | 0.17 | 0 | 0.93 | 1 | 1043 |
| 14 | 0.72 | 0.84 | 0.36 | 45 | 5166 |
| 15 | 0.52 | 0 | 1 | 9 | 1747 |
| 16 | 0.86 | 0.77 | 0.8 | 64 | 3220 |
| 17 | 0.57 | 0.33 | 0.8 | 15 | 3996 |
| 18 | 0.89 | 0.7 | 0.86 | 10 | 702 |
| 19 | 0.83 | 0.64 | 0.84 | 137 | 4260 |
| 20 | 0.6 | 0.27 | 0.89 | 11 | 1581 |
| 21 | 0.63 | 0.18 | 0.85 | 17 | 929 |
| 22 | 0.89 | 0.72 | 0.9 | 46 | 2124 |

Table S1b

| Chromosome | AUC | TP | TN | High | Low |
| --- | --- | --- | --- | --- | --- |
| 1 | 0.95 | 0.95 | 0.84 | 442 | 2052 |
| 2 | 0.93 | 0.96 | 0.79 | 328 | 1247 |
| 3 | 0.94 | 0.97 | 0.81 | 275 | 1115 |
| 4 | 0.92 | 0.95 | 0.78 | 128 | 616 |
| 5 | 0.94 | 0.94 | 0.83 | 226 | 735 |
| 6 | 0.93 | 0.95 | 0.78 | 240 | 887 |
| 7 | 0.94 | 0.96 | 0.81 | 263 | 1145 |
| 8 | 0.93 | 0.93 | 0.79 | 213 | 725 |
| 9 | 0.94 | 0.94 | 0.81 | 239 | 910 |
| 10 | 0.94 | 0.93 | 0.87 | 175 | 800 |
| 11 | 0.92 | 0.95 | 0.78 | 365 | 1314 |
| 12 | 0.95 | 0.96 | 0.85 | 267 | 1107 |
| 13 | 0.89 | 0.91 | 0.79 | 81 | 276 |
| 14 | 0.94 | 0.95 | 0.84 | 129 | 1071 |
| 15 | 0.95 | 0.93 | 0.83 | 147 | 722 |
| 16 | 0.95 | 0.95 | 0.87 | 287 | 1332 |
| 17 | 0.93 | 0.92 | 0.84 | 359 | 1573 |
| 18 | 0.96 | 0.98 | 0.87 | 65 | 236 |
| 19 | 0.9 | 0.93 | 0.76 | 549 | 1771 |
| 20 | 0.93 | 0.95 | 0.81 | 173 | 624 |
| 21 | 0.95 | 0.94 | 0.88 | 47 | 291 |
| 22 | 0.95 | 0.98 | 0.78 | 155 | 788 |

Table S1c

| Chromosome | AUC | TP | TN | High | Low |
| --- | --- | --- | --- | --- | --- |
| 1 | 0.83 | 0.63 | 0.87 | 1740 | 15180 |
| 2 | 0.81 | 0.57 | 0.92 | 833 | 11092 |
| 3 | 0.81 | 0.64 | 0.86 | 953 | 8100 |
| 4 | 0.81 | 0.64 | 0.88 | 287 | 5835 |
| 5 | 0.83 | 0.62 | 0.92 | 636 | 6498 |
| 6 | 0.85 | 0.69 | 0.89 | 849 | 7589 |
| 7 | 0.82 | 0.6 | 0.9 | 605 | 7251 |
| 8 | 0.83 | 0.61 | 0.9 | 543 | 4813 |
| 9 | 0.85 | 0.66 | 0.92 | 656 | 6467 |
| 10 | 0.76 | 0.49 | 0.92 | 621 | 6560 |
| 11 | 0.83 | 0.69 | 0.85 | 817 | 8167 |
| 12 | 0.8 | 0.59 | 0.88 | 968 | 7488 |
| 13 | 0.87 | 0.72 | 0.91 | 179 | 2831 |
| 14 | 0.91 | 0.69 | 0.95 | 531 | 10516 |
| 15 | 0.85 | 0.62 | 0.92 | 481 | 5551 |
| 16 | 0.86 | 0.66 | 0.91 | 674 | 5904 |
| 17 | 0.84 | 0.65 | 0.88 | 1034 | 7325 |
| 18 | 0.82 | 0.62 | 0.93 | 144 | 2504 |
| 19 | 0.85 | 0.79 | 0.77 | 962 | 7637 |
| 20 | 0.81 | 0.59 | 0.85 | 445 | 3583 |
| 21 | 0.83 | 0.58 | 0.91 | 120 | 1813 |
| 22 | 0.76 | 0.63 | 0.8 | 239 | 3757 |

Table S1d

| Chromosome | AUC | TP | TN | High | Low |
| --- | --- | --- | --- | --- | --- |
| 1 | 0.71 | 0.71 | 0.57 | 66 | 10840 |
| 2 | 0.85 | 0.53 | 0.9 | 53 | 8584 |
| 3 | 0.78 | 0.58 | 0.85 | 113 | 6539 |
| 4 | 0.91 | 0.81 | 0.81 | 36 | 4643 |
| 5 | 0.87 | 0.7 | 0.87 | 135 | 5137 |
| 6 | 0.86 | 0.78 | 0.78 | 106 | 5599 |
| 7 | 0.51 | 0.06 | 0.92 | 16 | 5251 |
| 8 | 0.78 | 0.65 | 0.82 | 51 | 3729 |
| 9 | 0.83 | 0.63 | 0.88 | 41 | 4832 |
| 10 | 0.76 | 0.73 | 0.69 | 83 | 4825 |
| 11 | 0.87 | 0.85 | 0.76 | 92 | 5546 |
| 12 | 0.89 | 0.83 | 0.81 | 80 | 5492 |
| 13 | 0.91 | 0.73 | 0.92 | 26 | 2257 |
| 14 | 0.9 | 0.63 | 0.9 | 35 | 6595 |
| 15 | 0.87 | 0.82 | 0.83 | 55 | 4224 |
| 16 | 0.76 | 0.42 | 0.83 | 26 | 3784 |
| 17 | 0.85 | 0.75 | 0.76 | 76 | 4678 |
| 18 | 0.89 | 0.86 | 0.67 | 28 | 1890 |
| 19 | 0.86 | 0.84 | 0.76 | 32 | 4208 |
| 20 | 0.84 | 0.79 | 0.81 | 28 | 2310 |
| 21 | 0.9 | 0.71 | 0.84 | 7 | 1298 |
| 22 | 0.87 | 0.5 | 0.93 | 6 | 2095 |
