# Supplementary material for: Occupancy Classification of Position Weight Matrix-Inferred Transcription Factor Binding Sites
Source: PLoS One. 2011 Nov 4;6(11):e26160. doi: 10.1371/journal.pone.0026160 (PMC3208542; doi:10.1371/journal.pone.0026160)
Supplement: Table S2 — A: Top 10 most frequently occurring predictors in the occupancy classifiers (per-TF and cumulative). B: Average class-conditional probability of high-occupancy status for smallest distance bin for top 10 most frequently occurring features in cross-classification (two-bin cases only). C: Average class-conditional probability of high-occupancy status for smallest distance bin for top 10 most frequently occurring features per-chromosome(all cases). (DOC) [file pone.0026160.s002.doc]

Supplementary Table S2

Supplementary Table 2Sa

| Predictor | GABP | c-Myc | STAT | TCF4 | Total |
| --- | --- | --- | --- | --- | --- |
| H3K4me2-H3K4me3 | 80 | 8 | 83 | 57 | 228 |
| H3K27me1-H3K4me3 | 84 | 11 | 87 | 43 | 225 |
| TSS-H3K4me3 | 85 | 47 | 50 | 22 | 204 |
| H3K79me1-H3K4me3 | 69 | 26 | 79 | 25 | 199 |
| H3K79me2-H3K4me3 | 97 | 12 | 55 | 34 | 198 |
| H4R3me2-H3K4me3 | 61 | 40 | 74 | 23 | 198 |
| H3K79me2-TSS | 81 | 44 | 51 | 20 | 196 |
| H4K20me1-H3K4me3 | 68 | 10 | 82 | 30 | 190 |
| H3K79me3-H3K4me3 | 30 | 36 | 72 | 49 | 187 |
| H3K9me1-H3K4me3 | 78 | 10 | 77 | 13 | 178 |

Supplementary Table 2b:

| Feature | c-Myc | GABP | STAT | TCF4 |
| --- | --- | --- | --- | --- |
| H3K4me2-H3K4me3 | 0.65 | 0.91 | 0.59 | 0.54 |
| H3K27me1-H3K4me3 | 0.38 | 0.91 | 0.52 | 0.42 |
| TSS-H3K4me3 | 0.82 | 0.97 | 0.78 | 0 |
| H3K79me1-H3K4me3 | 0.63 | 0.87 | 0.58 | 0.46 |
| H3K79me2-H3K4me3 | 0.49 | 0.88 | 0.55 | 0.42 |
| H4R3me2-H3K4me3 | 0.3 | 0 | 0.54 | 0.43 |
| H3K79me2-TSS | 0.6 | 0.93 | 0.7 | 0.6 |
| H4K20me1-H3K4me3 | 0.28 | 0.91 | 0.49 | 0.38 |
| H3K79me3-H3K4me3 | 0.86 | 0.82 | 0.57 | 0.59 |
| H3K9me1-H3K4me3 | 0.38 | 0.92 | 0.54 | 0 |

Supplementary Table S2c:

| Feature | c-Myc | GABP | STAT | TCF4 |
| --- | --- | --- | --- | --- |
| H4K20me1-H3K4me3 | 0.79 | 0.91 | 0.58 | 0.67 |
| H3K4me2-H3K4me3 | 0.6 | 0.93 | 0.57 | 0.7 |
| H3K79me3-H3K4me3 | 0.81 | 0.92 | 0.53 | 0.72 |
| TSS-H3K4me3 | 0.94 | 0.98 | 0.67 | 0 |
| H3K79me2-H3K4me3 | 0.43 | 0.91 | 0.56 | 0.57 |
| H3K27me1-H3K4me3 | 0.66 | 0.91 | 0.57 | 0.5 |
| H3K79me1-H3K4me3 | 0.93 | 0.91 | 0.55 | 0.72 |
| H3K79me2-TSS | 0.92 | 0.87 | 0.68 | 0.6 |
| H4R3me2-H3K4me3 | 0 | 0.82 | 0.52 | 0.74 |
| H3K9me1-H3K4me3 | 0.68 | 0.92 | 0.47 | 0.56 |
